# A Review of the Biological Activities of Microalgal Carotenoids and Their Potential Use in Healthcare and Cosmetic Industries

**DOI:** 10.3390/md16010026

**Published:** 2018-01-12

**Authors:** Ramaraj Sathasivam, Jang-Seu Ki

**Affiliations:** Department of Biotechnology, Sangmyung University, Seoul 03016, Korea; ramarajbiotech@gmail.com

**Keywords:** carotenoids, microalgae, anti-angiogenic, cardioprotective activity, anti-cancer, anti-diabetic, anti-inflammatory, anti-obesity, anti-oxidant, beauty

## Abstract

Carotenoids are natural pigments that play pivotal roles in many physiological functions. The characteristics of carotenoids, their effects on health, and the cosmetic benefits of their usage have been under investigation for a long time; however, most reviews on this subject focus on carotenoids obtained from several microalgae, vegetables, fruits, and higher plants. Recently, microalgae have received much attention due to their abilities in producing novel bioactive metabolites, including a wide range of different carotenoids that can provide for health and cosmetic benefits. The main objectives of this review are to provide an updated view of recent work on the health and cosmetic benefits associated with carotenoid use, as well as to provide a list of microalgae that produce different types of carotenoids. This review could provide new insights to researchers on the potential role of carotenoids in improving human health.

## 1. Introduction

Carotenoids have a wide range of applications in the healthcare and nutraceuticals industry. The growing importance of carotenoid use in improving food quality has also led to an increase in the demand for carotenoids in the global market. The global carotenoid market was estimated to be ~1.24 billion USD in 2016, and is projected to increase to ~1.53 billion USD by 2021, at a compound annual growth rate (CAGR) of 3.78% from 2016 to 2021 (www.bccresearch.com). Due to changes in life style and the rising health consciousness of the average population, the demand for nutrient-rich supplements with health benefits (such as immunity boosters and supplements rich in proteins and vitamins) has risen. Carotenoids have various medicinal properties and they are widely used as preventives against diseases such as cancer, diabetes, and cataract [1]. Carotenoids are also used in food supplements, cosmetics, and pharmaceuticals. Therefore, the use of carotenoids in supplements is likely to increase over the next few years. The major types of carotenoids that are used commercially in the global market are astaxanthin, β-carotene, lutein, canthaxanthin, lycopene, and zeaxanthin. Among these, β-carotene is the pigment carotene, which imparts a red, yellow, or orange color to plants, algae, and animals [2,3]. For this reason, production of carotenoids is considered as an important business opportunity for the healthcare and cosmetic industries in the future. As a result, many multinational companies have begun producing various types of carotenoids for use in different applications.

Currently, the bulk of industry-produced carotenoids are synthesized chemically, though a small portion of carotenoids is obtained naturally from plants or algae. Due to the adverse side effects commonly associated with drug therapy, public interest in recent times has focused on natural products with health-promoting properties as alternatives to conventional drugs. There is a growing demand for natural compounds because of an increasing global trend in the use of, and consumer preference for products made with natural ingredients. Since algae represent an alternative source of natural carotenoids, carotenoid extraction from cultivated algae may help in overcoming problems with balancing the supply of and demand for these products [4].

Microalgae are photosynthetic microorganisms, which may be widely used as a potential source for the production of several highly valuable bioproducts. There are ~13,000 species, of which ~8000 are described, and ~5000 species are yet to be described [5]. Microalgae are a rich source of bioactive compounds such as vitamins, proteins with essential amino acids, polysaccharides, fatty acids, minerals, photosynthetic pigments (carotenoids and chlorophylls), enzymes, and fiber [6,7,8]. Due to the high levels of biologically valuable components in microalgae, these organisms can represent a good source of supplements with various health benefits. Foods rich in chlorophyll are considered to be very nutritious for humans as they contain high levels of heme and can help in increasing the production of red blood cells [9]. In addition, consumption of microalgae has recently been shown to provide health benefits; microalgae can therefore function as nutraceutical agents with antioxidant, anti-inflammatory, anti-mutagenic, and anti-microbial properties [10].

Reviews on the benefits of carotenoids for human health, and the application-based uses of carotenoids ([11,12], respectively), unfortunately, do not provide much information on the uses of carotenoids in the healthcare and beauty industries. In addition to these reviews, a review by Raposo et al. [13] elaborates the use of marine microalgal carotenoids for the prevention of chronic diseases; another study by Di Pietro et al. [14] summarizes the use of carotenoids in cardiovascular disease prevention. Recently, there has been a rising interest in exploring the beneficial effects of carotenoids in the healthcare and cosmetics industries. Therefore, the main aim of this article is to critically review available data on the use of carotenoids, and their mechanisms of action in providing healthcare and cosmetic benefits. We will summarize the anti-angiogenic, cardioprotective activity anti-cancer, anti-diabetic, anti-inflammatory, anti-obesity, anti-oxidant, beauty-enhancing and other beneficial effects of carotenoids in this article. We hope this review will further improve our understanding of carotenoids and their therapeutic potential in healthcare and for cosmetic benefits.

## 2. Types of Carotenoids

The two main groups of carotenoids are carotenes and xanthophylls. Some familiar carotenes are β-carotene and lycopene—both these carotenoids are strict hydrocarbon carotenoids, and do not possess any substituent (or even oxygen) in their structures. Xanthophylls or oxycarotenoids, which belong to the second group, are oxygen-containing molecules. Lutein and zeaxanthin are two xanthophylls with –OH groups in their structures, whereas canthaxanthin and echinenone contain =O groups. Astaxanthin has both –OH and =O groups in its structure. Furthermore, some carotenoids such as violaxanthin and diadinoxanthin contain epoxy groups, and others such as dinoxanthin and fucoxanthin have acetyl groups in their structures. The two carotenoids with acetyl groups also contain the C=C=C (allene) group in their structures, which is unique to natural products [15]. In addition, some carotenoids such as allo-, diato-, diadino-, hetero-, croco-, pyro-, and monadoxanthin contain C≡C (acetylene) groups in their structures. The different types of carotenoids mentioned above are naturally produced by the microalgae showed in Table 1.

## 3. Functions in Algal Cells

In plants, carotenoids play a significant role in the photosynthetic process by forming pigment–protein complexes in the thylakoid membrane; in cyanobacteria, however, they may also be found in the plasma membrane, and function in protecting the cells during exposure to high light intensities [52,53]. In algae, carotenoids protect chlorophyll from the effects of excess light exposure (by scavenging reactive oxygen species (ROS) such as singlet oxygen molecules and free radicals [54]) and are also required for phototropism and phototaxis [55] as they are present in algal eyespots [56]. Previous studies have reported that β-carotene also plays a role in protecting cells from free radicals, and that other carotenoids are mainly involved in light-harvesting functions [57]. In addition, fucoxanthin in algae acts as a primary light-harvesting carotenoid that transfers energy to chlorophyll–protein complexes. The fucoxanthin molecule exhibits high energy transfer efficiency (~80%), a property that is attributed to the unique structure of this carotenoid [58]. Fucoxanthin also participates in photoprotection and has strong antioxidant activity [40,59]. In addition, under unfavorable conditions, certain microalgae defend themselves by producing secondary metabolites via the carotenogenesis pathway [20]. To date, little information is available in the public database about the genes and enzymes involved in the algal carotenogenesis pathway [60].

## 4. Molecular Details of the Carotenoid Pathway in Microalgae

Our current understanding of the carotenoid metabolic pathway and its regulation in microalgae is unclear, and is mainly inferred from our knowledge of the process in higher plants. In chloroplasts, several nucleus-encoded membrane proteins are involved in the synthesis of carotenoids. These proteins are produced in the cytoplasm as precursor polypeptides with amino-terminal extensions that target them to chloroplasts. There are many publications that comprehensively review the carotenoid biosynthetic pathway [61,62,63,64].

**Figure 1:**
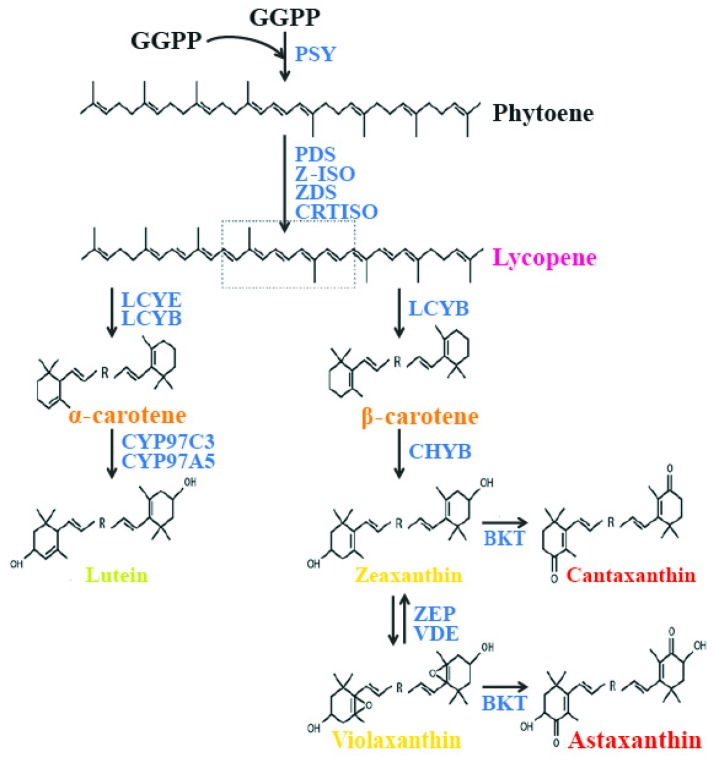
Carotenoid biosynthesis pathway in chlorophytes. GGPP—geranylgeranyl pyrophosphate; PSY—phytoene synthase; PDS—phytoene desaturase; Z-ISO—ζ-carotene isomerase; ZDS—ζ-carotene desaturase; CRTISO—carotene isomerase; LCYE—lycopene ε-cyclase; LCYB—lycopene β-cyclase; CYP97C3—cytochrome P450 ε-hydroxylase; CYP97A5—cytochrome P450 β-hydroxylase; CHYB—carotene β-hydroxylase; BKT—β-carotene oxygenase; ZEP—zeaxanthin epoxidase; VDE—violaxanthin de-epoxidase.

Carotenoids are synthesized from a C5 building block, a common precursor to all isoprenoids, which is the isopentenyl pyrophosphate (IPP) molecule, via the plastidial 2-C-methyl-d-erythriol 4-phosphate (MEP) pathway [65]. The first step in carotenoid biosynthesis is the condensation of two molecules of geranyl geranyl pyrophosphate (GGPP), catalyzed by the enzyme phytoene synthase (PSY), to yield phytoene, the first but uncolored carotenoid. Following this step, phytoene undergoes a series of sequential desaturations catalyzed by phytoene desaturase (PDS) and ζ-carotene desaturase (ZDS), which result in the formation of pro-lycopene. Pro-lycopene is then isomerized by a specific carotenoid isomerase (CRTISO) into all-trans lycopene. After this step, the pathway is divided into two branches. In one branch of the synthetic pathway, lycopene is cyclized at both ends by lycopene β-cyclase (LCYB), yielding β-carotene with two β–ionone end groups. These can be further hydroxylated by a non-heme di-iron hydroxylase, called β-carotene hydroxylase (CHYB), to yield zeaxanthin. In the other branch of the synthetic pathway, the combined actions of LCYB and lycopene ε-cyclases (LCYEs) result in the formation of α-carotene. The quantities of carotenoids produced by each branch of the pathway are determined by the absolute activities of LCYE and LCYB. The hydroxylation of α-carotene is catalyzed by two heme-containing cytochrome P450 monoxygenases (namely, carotene β-hydroxylase and carotene ε-hydroxylase), which leads to the formation of lutein. In another branch, zeaxanthin is converted to violaxanthin by zeaxanthin epoxidase (ZEP), which inserts two epoxy groups at positions C-5,6 and C-5′,6′ [62]. In another branch of the pathway, zeaxanthin is converted to the di-keto carotenoid canthaxanthin, and violaxanthin is converted to astaxanthin by the enzyme β-carotene ketolase (BKT) (Figure 1).

In most microalgae, the basic carotenoid synthesis pathways are highly conserved, although some species are able to accumulate unusual carotenoids via specific biosynthetic routes. In higher plants, most of the genes encoding enzymes for the carotenoid biosynthetic pathway have been isolated and characterized by functional complementation experiments [66]. In microalgae, most of the carotenoid biosynthetic pathway genes have also been isolated and characterized [67].

## 5. Carotenoids and Their Biological Activities

### 5.1. Anti-Angiogenic Activity

Angiogenesis is the process of formation of new blood vessels from pre-existing capillaries and involves a sequence of events that are fundamental to many physiological and pathological processes [68]. It occurs throughout life, during both healthy and diseased conditions, and is tightly regulated under normal physiological conditions such as during embryogenesis, ovary cycling, and wound healing. Chronic, unregulated angiogenesis can lead to several anomalous angiogenic conditions such as inflammatory diseases, rheumatoid arthritis, and tumor metastasis [69]. Tumor growth and metastasis are processes that are highly dependent on the formation of new blood vessels. Therefore, preventing angiogenesis under pathological conditions (such as cancer and other angiogenesis related diseases) is a promising approach for controlling or eradicating such diseases.

Studies involving in vivo and in vitro experiments on male C57BL/6 mice and B16F-10 cells have been used to evaluate the anti-angiogenic effects of β-carotene by Guruvayoorappan and Kuttan [70]. Their study found that treatment with β-carotene significantly reduces the number of tumor-directed capillaries (associated with altered serum cytokine levels) formed, and suppresses the proliferation, migration, and tube formation of endothelial cells. In addition, β-carotene treatment also inhibited the activation and nuclear translocation of p65, p50, and c-Rel sub-units of nuclear factor-κB (NF-κB), as well as other transcription factors such as c-fos, activated transcription factor-2, and cyclic adenosine monophosphate response element-binding protein in B16F-10 melanoma cells [70]. This study clearly showed that the anti-angiogenic effect of β-carotene occurs by affecting serum cytokine levels, and that β-carotene could inhibit the activation and nuclear translocation of transcription factors.

**Figure 2:**
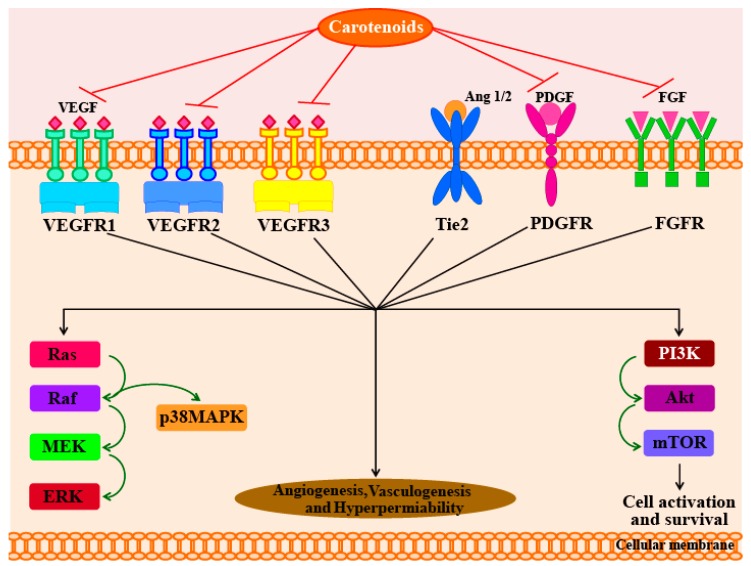
Diagrammatic representation of anti-angiogenic effect of carotenoid. Both VEGF and non-VEGF dependent pathways are noted. Akt—protein kinase B; ERK—extracellular signal-reduced kinase; FGF—fibroblast growth factor; FGFR—fibroblast growth factor receptor; MEK—mitogen-activated protein kinase/extracellular signal-reduced kinase kinase; mTOR—mechanistic target of rapamycin; p38 MAPK—p38 mitogen-activated protein kinase; PDGF—platelet-derived growth factor; PDGFR—platelet-derived growth factor receptor; PI3K—phosphoinositide 3-kinase; Tie2—tyrosine kinase with immunoglobulin-like and EGF-like domains 1; RAF—rapidly accelerated fibrosarcoma; RAS—rat sarcoma; VEGF—vascular endothelial growth factor; VEGFR—vascular endothelial growth factor receptor.

In another study, Sugawara et al. [71] reported that fucoxanthin (at concentrations of >10 µM) can significantly inhibit tube formation and proliferation in human umbilical vein endothelial cells (HUVECs). Fucoxanthin significantly suppressed the differentiation of endothelial progenitor cells into endothelial cells during the formation of new blood vessel. Fucoxanthin and its metabolite fucoxanthinol also suppressed the growth of microvessels during in vitro and ex vivo experiments using rat aortic rings [71]. In addition, a study in 2013 used HUVECs to understand the molecular mechanisms responsible for the anti-angiogenic activity of fucoxanthin [72]. The results of this study showed that fucoxanthin significantly reduced the genetic expression, and hence, mRNA levels of fibroblast growth factor 2 (FGF-2), its receptor (FGFR-1), as well as their trans-activation factor, EGR-1. However, the study found no significant changes in the mRNA levels of the vascular endothelial growth factor receptor-2 (VEGFR-2). Furthermore, fucoxanthin was found to down-regulate the phosphorylation of FGF-2-mediated intracellular signaling proteins such as extracellular signal-reduced kinase and protein kinase B (ERK1/2 and Akt). Matrigel invasion assays showed that fucoxanthin not only inhibited the migration of endothelial cells, but also inhibited their differentiation into tube-like structures by suppressing the phosphorylation of the FGF-2-mediated intracellular signaling proteins. However, there was no evidence to indicate that carotenoids activate the angiopoietins 1 and 2 (Ang1 and Ang2) pathways. The possible molecular mechanisms responsible for the anti-angiogenic effects of carotenoids are shown in Figure 2.

### 5.2. Cardioprotective Activity

A study in 2005 by Hussein et al. [73] reported the anti-hypertensive effects of astaxanthin in spontaneously hypertensive rats (SHRs). In their study, Hussein et al. [73] found that oral administration of astaxanthin (at a concentration of 50 mg/kg) for 14 days led to a significant decrease in blood pressure in the SHRs. In addition, long-term administration of astaxanthin (for 5 weeks) also considerably reduced blood pressure and postponed the occurrence of heart strokes in these rats. On the 4th day of treatment, 60% of the rats in the placebo group showed signs of heart stroke, whereas none of the rats in the astaxanthin-treated group showed any signs of heart stroke. In later studies, the same authors also reported the mechanism of how astaxanthin works to prevent heart strokes [74]. The authors found that SHRs treated with astaxanthin showed significantly higher levels of vasorelaxation in response to nitric oxide (NO), which enhanced thoracic aorta contractions, as compared to rats not treated with astaxanthin. These results suggest that the anti-hypertensive effect of astaxanthin is mediated by NO-related mechanisms. In addition, another study carried out on SHRs to explore the beneficial effects of astaxanthin on blood rheology found that the transit times of whole blood in astaxanthin-treated SHRs were significantly lower than those of placebo-treated SHRs. Histopathological measures, such as levels of vascular elastin in the aorta and arterial wall thickness were also improved in SHRs treated with astaxanthin [73,74]. A study by Preuss et al. [75] in Zucker fatty rats found that administration of 25 mg/kg of astaxanthin for one month significantly lowered systolic blood pressure. In addition, the astaxanthin treatment also decreased the activity of the renin-angiotensin system, which indicates that the lowering in blood pressure was dependent on changes in the renin-angiotensin, as well as the NO systems. Furthermore, in heat stress experiments, all rats fed with astaxanthin survived, whereas, >60% of the rats in the placebo group died.

In another study, female BALB/c mice treated with 800 mg/kg astaxanthin for eight weeks exhibited higher heart mitochondrial membrane potentials and contractility indices than mice in a placebo group [76]. An ex vivo study of 24 adult humans showed that astaxanthin has the potential to prevent atherosclerosis by delaying the prolonged oxidation of low-density lipoprotein (LDL)-cholesterol. In this study, volunteers consumed astaxanthin at doses of 1.8, 3.6, 14.4, or 21.6 mg/day for 14 days and the LDL lag times were longer (5.0%, 26.2%, 42.3%, and 30.7%, respectively) compared with the initial day [77]. Experiments by Miyawaki et al. [78] to determine the health benefits of astaxanthin extracted from *Haematococcus pluvialis* on human blood rheology were carried out on 20 adult men. After 10 days of astaxanthin (6 mg/day) administration, the whole blood transit time of the experimental group decreased from 52.8 ± 4.9 s to 47.6 ± 4.2 s, which is considerably lower than that of the control group (54.2 ± 6.7 s) [78]. In addition, another study carried out on humans in an age group of 25–60 years [79] showed that 12 weeks of astaxanthin administration significantly decreased serum triglyceride levels, while significantly increasing high density lipoprotein (HDL)-cholesterol levels. However, LDL-cholesterol levels remained unchanged. Furthermore, astaxanthin intake increased serum adiponectin levels, which are positively correlated with changes in HDL-cholesterol levels independent of age and body mass index (BMI) [79]. Fucoxanthin and its derivative fucoxanthinol show cardioprotective activity; administration of these carotenoids in an in vivo study reduced triglyceride levels in blood (high triglyceride levels in blood are related to the development of atherosclerotic vascular disease) [80]. When rats were fed with 2 mg/kg of fucoxanthin or fucoxanthinol, they showed a significant reduction in triglyceride absorption in their jugular veins on being fed with non-pre-digested 10% soybean oil.

### 5.3. Anti-Cancer Activity

Numerous in vitro and in vivo studies have demonstrated the anti-cancer activities of carotenoids. The results of these studies indicate that carotenoids may prevent different types of cancers in humans, including bladder, breast, hepatic, intestinal, leukemic, lung, oral, and prostate cancer. The anti-cancer activity of carotenoids involves a variety of mechanisms, including induction of cell apoptosis and suppression of cell proliferation. In particular, one in vivo study showed that β-carotene, astaxanthin, canthaxanthin, and zeaxanthin help in reducing the sizes and numbers of liver neoplasias [51]. Another study also reports that dietary intake of carotenoids can reduce the risk of developing colon cancer [81,82].

Many studies indicate that β-carotene shows great potential as an anti-tumor agent. In a study in China, administration of a combination of β-carotene, vitamin E, and selenium to humans was observed to decrease the incidence of mortality due to cancer [83]. Many other studies have also reported an inverse relationship between ingesting carotenoids and cancer prevalence [84,85]. Lycopene is one of the best studied carotenoids with respect to its potential health benefits [86,87]; this is because it exhibits much higher anti-cancer potential than most other carotenoids [51]. Several in vivo and in vitro studies using tumor cell lines indicate that lycopene can significantly reduce tumor cell growth [86,87]. Nishino et al. [51] have reported that the carotenoids α-carotene, lutein, zeaxanthin, lycopene, β-cryptoxanthin, fucoxanthin, astaxanthin, capsanthin, crocetin, and phytoene exhibit greater anti-carcinogenic activity than β-carotene.

**Figure 3:**
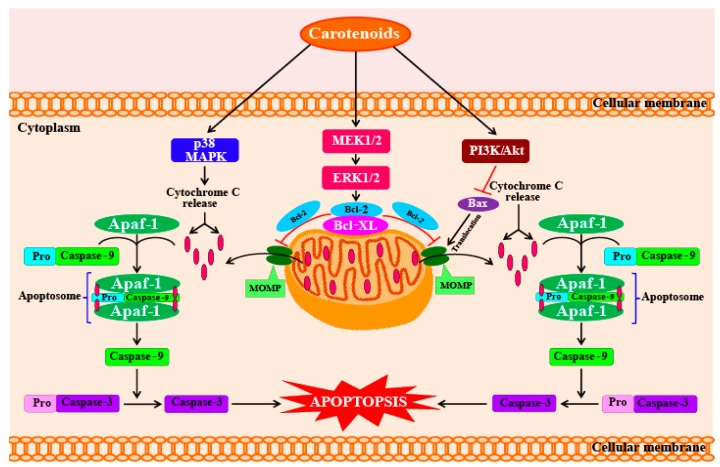
Factors (molecules and mechanisms) regulated by carotenoids, resulting in their anti-carcinogenic effects. Carotenoids induce the activation of PI3K/Akt survival pathway, trigger the phosphorylation-dependent inactivation of Bax (Bcl-2 associated X), which leads to apoptosis by decrease in caspase activity. Carotenoids also maintain the mitochondrial integrity by regulating the p38 MAPK signaling pathway, which leads to a decrease in cytochrome c release and inhibits caspase-dependent apoptotic cell death. APAF-1—Apoptotic protease activating factor-1; BAX—Bcl-2 associated X; Bcl-2—B-cell lymphoma 2; Bcl-XL—B-cell lymphoma-extra-large; ERK—extracellular signal-reduced kinase; MEK—mitogen-activated protein kinase/extracellular signal-reduced kinase kinase; MOMP—mitochondrial outer membrane permeabilization; p38 MAPK—p38 mitogen-activated protein kinase; PI3K—phosphoinositide 3-kinase.

The anti-proliferative and cancer-preventive activities of fucoxanthin and fucoxanthinol are dependent on different molecules and pathways involved in the processes of cell cycle arrest, apoptosis, and metastasis [88]. Furthermore, studies using human umbilical vein endothelial cells (HUVECs) have shown that fucoxanthin also has anti-angiogenic activity, which is helpful in preventing cancer. The detailed mechanisms of how fucoxanthin functions in this respect are explained in Section 5.1. Fucoxanthin can potentially inhibit the proliferation of cancer cells by increasing intercellular communication through gap junctions in human cancer cells, which increases intracellular signaling mechanisms that promote cell cycle arrest and apoptosis. Therefore, fucoxanthin and its metabolites show great potential as chemotherapy agents if administered in the initial stages of cancer [88]. In addition, fucoxanthin also lowers the viabilities of human leukemia (HL-60) cells. Fucoxanthin also shows anti-cancer activity against Caco-2, DLD-1, and HT-29, which are human colorectal adenocarcinoma cell lines. Although fucoxanthin treatment has been shown to reduce cell viability, the strength of the effect varies across cell types. After 72 h of fucoxanthin treatment (at a concentration of 15.2 mM), the viabilities of Caco-2, DLD-1, and HT-29 cells decreased to 14.8%, 29.4%, and 50.8%, respectively [89]. These remarkable reductions in cell viability levels were caused by a significant increase in cell apoptosis and DNA fragmentation [89]. Kim et al. [90] reported that astaxanthin, β-carotene, and fucoxanthin show potent anti-cancer activities when tested on HL-60 cancer cells at a concentration of 7.6 mM. At this concentration, fucoxanthin caused high levels of DNA fragmentation, whereas the other two carotenoids (astaxanthin and β-carotene) did not show any significant effects on DNA fragmentation. Kim et al. [90] stated that the mechanism of fucoxanthin-induced apoptosis in HL-60 cells involves the generation of ROS, which leads to cytotoxicity and apoptosis involving the cleavage of caspases-3 and -9 and poly-ADP-ribose polymerase (PARP), coupled with reductions in levels of Bcl-xL (Figure 3). Kotake-Nara et al. [91] investigated the effects of fucoxanthin (at concentrations of 5 and 10 mM) on the viabilities of six types of cancer cells. Incubation with fucoxanthin for 72 h showed that five of the cancer cell lines suffered significant reductions in cell viability. In addition, comparisons of the effects of fucoxanthin and lycopene on cancer cells indicate that at the same concentrations, fucoxanthin shows higher anti-cancer effects than lycopene. Fucoxanthin is a potential chemopreventive agent for urinary bladder cancers, as it inhibits the growth and causes apoptosis in EJ-1 cells (a urinary bladder cancer cell line). Treatment with fucoxanthin significantly reduced EJ-1 cell proliferation in a dose- and time-dependent manner. Treatment with 20 mM fucoxanthin for 72 h caused a high percentage of cells to undergo apoptosis (93%), which was evident by morphological changes, DNA fragmentation, increased percentages of hypodiploid cells, and caspase-3 activity [92].

The effect of different carotenoids such as β-cryptoxanthin, canthaxanthin, fucoxanthin, neoxanthin, phytoene, and zeaxanthin on the viabilities of three prostate cancer cell lines, namely, PC-3, DU 145, and LNCaP, has been investigated [93]. Among the carotenoids tested, fucoxanthin and neoxanthin exhibited the highest cell-growth inhibition rates; the other carotenoid showed no significant effects on cell viability. When compared to untreated control cells, the viabilities of cells treated with 20 mM fucoxanthin for 72 h were reduced to 14.9%, 5.0%, and 4.8% for PC-3, DU 145, and LNCaP cells, respectively [93]. In addition, another study investigating the anti-cancer activities of fucoxanthin and neoxanthin on PC-3 cells with an apoptosis assay [91] showed that treatment with 20 mM fucoxanthin for 48 h increased the percentage of apoptotic cells to >30%. This indicates that fucoxanthin induces apoptosis by activating caspase-3. Fucoxanthinol has also been found to induce apoptosis in PC-3 cells, and has a greater inhibitory effect on these cells as compared to fucoxanthin. The 50% inhibitory concentration (IC50) of fucoxanthin and fucoxanthinol on the proliferation of PC-3 cells was 3.0 and 2.0 mM, respectively [91]. A study to compare the effects of carotenoids such as β-carotene and astaxanthin, with those of xanthophyll carotenoids like fucoxanthin on human colon cancer cells [94] has showed that the xanthophyll carotenoid, fucoxanthin, has higher anti-cancer activity than the other carotenoids. Taken together, the study clearly showed that the fucoxanthin metabolites (halocynthiaxanthin and fucoxanthinol) have greater anti-cancer activities than fucoxanthin. However, the effects of these metabolites on cancer cells were highly variable depending on the types of cancer cells.

Canthaxanthin, which is another type of carotenoid with significant anti-cancer activity, has been reported to significantly inhibit the growth of JB/MS, B16F10 (a melanoma cell line), and PYB6 (a fibrosarcoma tumor cell line) cells at a concentration of 100 mM [95]. Treatment with canthaxanthin also induced apoptosis in WiDr (a human colon adenocarcinoma cell line) and SK-MEL-2 (a human melanoma cell line) cells by increasing the numbers of in situ nick-end labeled-positive nuclei [96]. Canthaxanthin induced apoptosis in both cell lines in a dose- and time-dependent manner. A study by Abdel-Fatth [97] found that treatment with 10 mM canthaxanthin for 48 h caused 18% and 20% of cells to undergo apoptosis in the WiDr and SK-MEL-2 cell lines, respectively. Furthermore, the growth of WiDr cells showed significantly higher inhibition than that of SK-MEL-2 cells. These results suggest that other pathways, such as stimulation of tumor necrosis factor-α (TNF-α) and other cytokines [97], or down-regulation of the epidermal growth factor receptor [98], are involved in the inhibitory effects of canthaxanthin on cancer cell lines. The effects of canthaxanthin on chemically induced mammary carcinogenesis in mice showed that dietary intake of canthaxanthin for three weeks prior to the induction of cancer with dimethylbenzanthracene could reduce the occurrence of cancer by 65% [99]. Another study reported that anti-cancer agents may have the ability to upregulate intercellular communication via gap junctions. Even at a low dosage of 1 mM, canthaxanthin increases the levels of connexin43 in C3H10T1/2 (mouse embryo) cells [100]. In addition to this, numerous studies on mice have investigated the effects of canthaxanthin on colon carcinogenesis [101], skin papillomas [102], and cervical cancer [103].

Several studies have reported that astaxanthin has significant anti-cancer effects on certain cancer types such as prostatic hyperplasias and prostatic cancers. Astaxanthin mainly inhibits the enzyme 5-α-reductase, which is involved in abnormal prostate growth [17,104]. The chemopreventive effect of these carotenoids against various cancer types has been extensively studied by Tanaka et al. [101]. In one study, the occurrence of colon cancer induced by azoxymethane in F344 rats was significantly lower in rats fed with 500 ppm astaxanthin or canthaxanthin for 34 weeks; furthermore, rats fed with these carotenoids had significantly lower multiplicity of neoplasms than those rats in the placebo group [101]. In addition, rats fed with carotenoids showed a significant reduction in cell proliferation activity and the development of aberrant crypt foci (ACF) in these rats was also observed to be inhibited [101]. The investigations of Tanaka et al. [105] on the effects of astaxanthin and canthaxanthin on mouse urinary bladder carcinogenesis revealed that lower incidences of pre-neoplastic lesions and neoplasms occurred in mice treated with 50 ppm astaxanthin or canthaxanthin for 20 weeks, as compared to the incidences of cancer in mice from the placebo group. In addition, it was also found that the number of silver-stained nucleolar organizer region proteins (AgNORs) in the transitional epithelium was reduced in the carotenoid-treated group. Furthermore, both carotenoids showed anti-proliferative effects on the cancer cells in the mouse urinary bladder, with astaxanthin showing higher levels of anti-proliferative activity as compared to canthaxanthin [105]. A study by Kozuki et al. [106] on the inhibitory activities of eight different carotenoids on AH109A cell-invasion showed that at concentrations ≥5 µM all carotenoids could significantly inhibit AH109A cell-invasion in a dose dependent manner. Among the carotenoids tested (which included canthaxanthin, astaxanthin, α-carotene, β-carotene, β-cryptoxanthin, lutein, lycopene, and zeaxanthin), canthaxanthin showed the highest effects on inhibiting the invasiveness of AH109A cells.

An investigation by Lyons and O’Brien [107] on the differential effects of algal extracts (containing 14% astaxanthin) and synthetic astaxanthin on cancer cells in culture showed that treatment with both, algal extracts and synthetic astaxanthin, can protect cells against UVA-induced DNA damage. In this study, it was found that 2 h of exposure to UVA could cause a significant increase in superoxide dismutase (SOD) activity, along with a marked decrease in glutathione (GSH) content in 1BR-3 cells. However, in cells pre-incubated with the algal extract (18 h prior to UVA exposure), there were no changes in the level of antioxidant enzymes even after UVA exposure. This result agrees with the result of another experiment, where intestinal cells treated with 10 mM astaxanthin were observed to maintain their GSH content, even after UVA exposure. In addition to these effects, astaxanthin has also been shown to inhibit prostate cancer cell proliferation in a dose-dependent manner by inducing androgen hormones [108]. Numerous in vivo studies have investigated the anti-cancer effects of astaxanthin. Jyonouchi et al. [109] observed that astaxanthin treatment can reduce the weights and sizes of tumors induced by transplantable methylcholanthrene-induced fibrosarcoma (Meth-A tumor) cells in mice. In addition, Kurihara et al. [110] reported that daily oral administration of astaxanthin to mice inhibited lipid peroxidation, which markedly attenuated the development of hepatic metastasis induced by restraint stress.

The anti-cancer properties of the carotenoid cryptoxanthin have not been investigated extensively. However, recent in vitro, in vivo, and human-intervention studies report that β-cryptoxanthin can differentially regulate the expression of P73 variants. In addition, these studies have found that this carotenoid has the ability to inhibit the proliferation of colon cancer cells and in conjunction with oxaliplatin, can induce apoptosis in cancer cells by negatively regulating ΔNP73 [111].

### 5.4. Anti-Diabetic Activity

Recent work on carotenoids suggests that these molecules may be more effective in treating and controlling diabetes than antioxidants. Studies have shown that levels of dietary carotenoids and concentrations of β-carotene in blood are inversely associated with fasting blood glucose levels and insulin resistance, respectively [112]. Numerous studies have reported that carotenoids reduce the risk of type 2 diabetes mellitus (T2DM) development in men and women [112,113]. It has also been observed that carotenoid intake is inversely related to HbA1c levels [114]. In addition, recent findings have confirmed that carotenoids such as lycopene, lutein, and zeaxanthin can protect against diabetic retinopathy [115].

Most studies on carotenoids and diabetes report the importance of carotenoids in dietary intake for the prevention and treatment of T2DM [116]. A recent study by Sugiura et al. [117] shows that in middle-aged and older Japanese patients, serum levels of α-carotene and β-cryptoxanthin are associated with lower incidences of T2DM. In addition, another study that investigated the interactions between serum concentrations of carotenoids and smoking with the incidence of diabetes mellitus over a time span of 15 years [118] showed that the incidence of T2DM is inversely associated with serum concentrations of carotenoids in nonsmokers. A similar result was also obtained by Ylonen et al. [112], who showed that serum concentrations of lutein, zeaxanthin, lycopene, α-carotene, and β-carotene were significantly lower in diabetic subjects. Most of these studies also report that there is an association between carotenoid intake and reductions in the risk of developing T2DM [113,118,119].

Astaxanthin, which is one of the best studied carotenoids, shows great potential in preventing and treating diabetes. Astaxanthin has higher antioxidant activity than other carotenoids such as lutein, β-carotene, and zeaxanthin [120], and can be consumed safely by humans [121]. In db/db mice (a well-known obesity model for T2DM), treatment with astaxanthin decreases glucose tolerance, enhances serum insulin levels, and attenuates blood glucose levels. These results indicate that astaxanthin has protective antioxidant effects that can help in the preservation of pancreatic β-cell function [122]. Bhuvaneswari et al. [123] have also reported similar anti-diabetic effects in high-fat, high-fructose diet HFFD mice. The effect of astaxanthin on metabolic syndrome has also been investigated in a rat experimental model. Astaxanthin was found to decrease blood glucose and triglyceride levels, as well as enhance serum levels of HDL-cholesterol and adiponectin [124]. Interestingly, recent studies have reported that astaxanthin primarily targets the peroxisome proliferator-activated receptor (PPARγ), which plays a pivotal role in carbohydrate metabolism. These studies also report that astaxanthin not only binds to PPARγ, but the carotenoid also affects the mRNA levels of this protein [125]. These results are consistent with another study that reports the anti-hyperglycemic effects of astaxanthin [126,127].

Another important carotenoid, β-carotene, has been investigated in detail regarding its usefulness in the treatment of diabetes. Hozumi et al. [128] reported a significantly inverse correlation between serum concentrations of β-carotene and serum levels of HbA1c in diabetic patients. Arnlov et al. [129] also reported that impaired insulin sensitivity is linked to low serum concentrations of β-carotene. In a study conducted by the European Prospective Investigation into Cancer and Nutrition–Netherlands (EPIC), investigations on 37,846 men and women revealed an inverse association between dietary intake of β-carotene and the risk of T2DM development [113], a result similar to that obtained by Coyne et al. [130] in a population-based study in Queensland, Australia. Furthermore, serum levels of β-carotene are reported to be important determinants of metabolic syndrome outcome [131]. Although β-carotene is well-studied with respect to its usefulness in preventing or treating diabetes, other carotenoids such as lutein have not been well investigated. Katyal et al. [132] found that lutein can lower streptozotocin (STZ)-induced hyperglycemia and shows significant antioxidant effects in the kidneys of diabetic rats.

**Figure 4:**
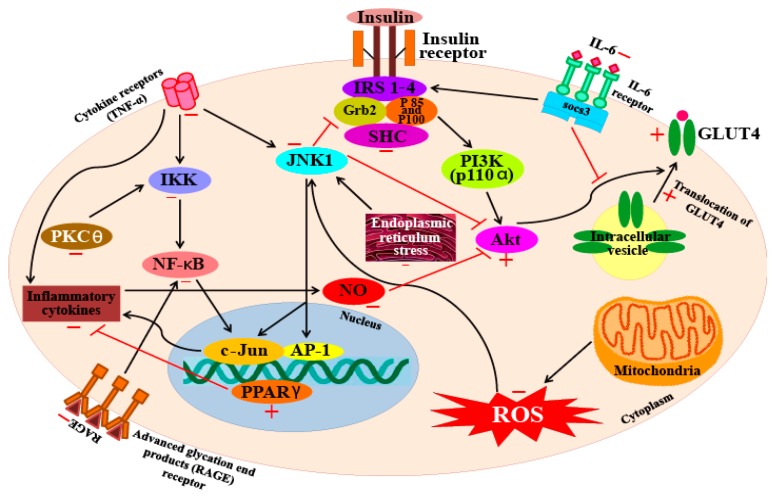
An overview on the targets of carotenoid with respect to their anti-diabetic effects. +, promote/activate; −, inactivate/inhibit. Akt—protein kinase B; AP1—activator protein 1 (c-jun and c-fos); GLUT 4—glucose transporter 4; Grb2—growth factor receptor-bound protein 2; IKK—IκB kinase; IL-6—interleukin-6; IRS 1–4—insulin receptor substrate 1–4; JNK 1—jun amino-terminal kinases 1; NF-κB—nuclear factor κB; NO—nitric acid; PI3K—phosphoinositide 3-kinase; PKCθ—protein kinase C θ; RAGE—receptor for advanced glycation end products; SHC—SH2-containing collagen-related proteins.

Another carotenoid, fucoxanthin, shows good potential as an anti-diabetic agent. A study on fucoxanthin reports that treatment with this carotenoid can restore blood glucose and insulin levels to normal in obese mice. The study reports that fucoxanthin upregulated the genetic expression and mRNA levels of the glucose transporter 4 (GLUT4) protein in skeletal muscle cells [133]. Nishikawa et al. [134] also report similar results (that fucoxanthin increases GLUT4 expression levels in skeletal muscle) and hypothesize that the induction of the PPARγ coactivator-1α mediates this process, which is accompanied by an upregulation in insulin receptor mRNA levels, along with increased phosphorylation levels of Akt, all of which play key roles in the regulation of GLUT4 translocation [134]. Maeda et al. [135] found that fucoxanthin significantly decreases the serum glucose and plasma insulin levels in diabetic/obese *KKAy* mice. Similarly, it has also been demonstrated that in *KKAy* mice, fucoxanthin can reduce hyperglycemia, although this carotenoid has no such effect on lean C57BL/6J mice [136]. Other studies have reported that the anti-diabetic activity of fucoxanthin involves several different mechanisms. For example, it has been demonstrated that fucoxanthin inhibits several enzymes such as aldose reductase in rat lens, human recombinant aldose reductase, protein tyrosine phosphatase 1B (PTP1B), and α-glucosidase, as well as processes such as advanced glycation end-product formation [137]. In addition, fucoxanthin has also been shown to increase the gene expression of PPARγ and GLUT4 proteins [135]. From these studies, it is clear that fucoxanthin manifests strong anti-diabetic effects through multiple mechanisms of action. Figure 4 illustrates the molecular targets involved in the anti-diabetic effects of carotenoids.

### 5.5. Anti-Inflammatory Activity

The first response of the immune system to infection or irritation is inflammation, which is also referred to as the innate cascade. However, some inflammatory reactions can have adverse effects on host cells or tissues; for example, chronic inflammation can cause many conditions such as arthritis, hepatitis, gastritis, periodontal disease, colitis, atherosclerosis, pneumonia, and neuro-inflammatory diseases [138]. Therefore, natural anti-inflammatory substances, especially carotenoids, are receiving much attention from researchers; carotenoids could potentially be used as drugs for preventing and controlling chronic inflammatory conditions due to their inhibitory effects on the production of NO, prostaglandin E_2_ (PGE_2_), and proinflammatory cytokines, as well as their inhibitory effects on enzymes such as inducible nitric oxide synthase (iNOS) and cyclooxygenase-2 (cox-2) [139,140].

Recently, astaxanthin has garnered much attention due to its potential as an anti-inflammatory agent. Both, in vitro and in vivo studies have been carried out in rat models to investigate the effects of astaxanthin on lipopolysaccharide (LPS)-induced inflammatory reactions [141,142]. The inhibitory effects of astaxanthin were compared with those of the common anti-inflammatory drug, prednisolone. The anti-inflammatory effect of astaxanthin (at a concentration of 100 mg/kg) was higher than that of 10 mg/kg of prednisolone [141]. LPS-fed mice treated with astaxanthin showed a dose-dependent anti-inflammatory effect; astaxanthin has been shown to function by suppressing the production of NO, PGE_2_, TNF-α, and interleukin-1β (IL-1β), as well as by blocking the activity of NOS enzymes in RAW 264.7 cells. The results of this study agree with those of a previously conducted study, which showed that astaxanthin inhibited NO production, as well as the expression of iNOS and COX-2 in LPS-stimulated BV2 microglial cells [143].

**Figure 5:**
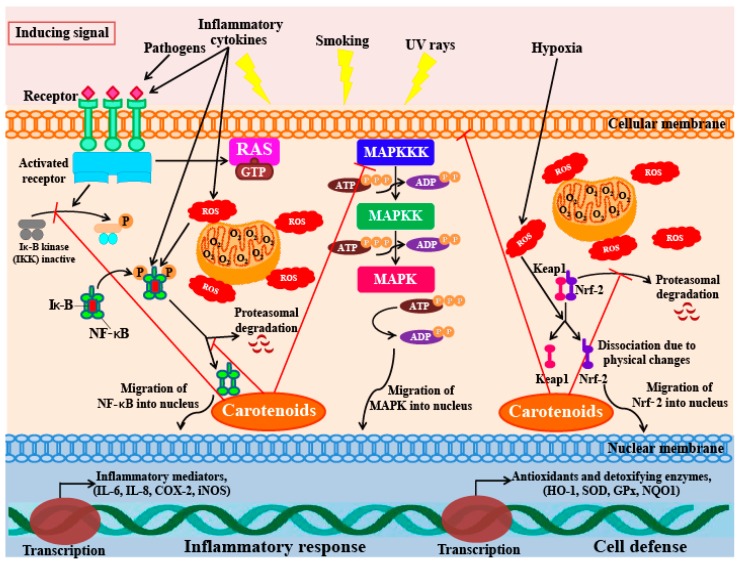
Schematic diagram of the interactions of anti-inflammatory signaling pathways and carotenoids. In the cytoplasm during the resting stage the NF-κB (nuclear factor-κB) is inactivated, which is bound to its inhibitory protein IκB (inhibitor of kappa B). During oxidative stress, inflammatory cytokines, or hypoxia, IκB protein is phosphorylated by the IKK (IκB kinase) complex, which leads to the ubiquitination and proteasomal degradation of IκB protein. At this time NF-κB was released which will migrate to the nucleus and the transcription of inflammatory mediators will start. It is assumed that carotenoids and their metabolites may interact with cysteine residues of the IKK and/or NF-κB subunits, which will inhibit the NF-κB pathway. In cytosol the Nrf2 (Nuclear factor (erythroid-derived 2)-like 2) is kept inactive by Keap1 (kelch-like ECH-associated protein 1) especially by poly-ubiquitination and rapid degradation through the proteasome. During redox imbalance, the Keap1-Nrf2 complex is disturbed, which leads to dissociation of Nrf2 from the complex. This Nrf2 migrates to the nucleus, which will induce the transcription of antioxidant and detoxifying enzymes, which promote cell protection. Carotenoids and their metabolites may interact with Keap1 by changing its physical properties. MAPK (mitogen-activated protein kinase) refers to a family of serine/threonine protein kinases. MAPK signaling cascades undergo consecutive and sequential step. MAPKs are phosphorylated and activated by MAPK-kinases (MAPKKs), which are further phosphorylated and activated by MAPKK-kinases (MAPKKKs). The MAPKKKs are in turn activated by interaction with small GTPases and/or other protein kinases family, which connect the MAPK module to cell surface receptors or external stimuli. However, it is still remains unclear how the carotenoids interact with the MAPK signaling pathway. COX—cyclooxygenase; GPx—glutathione peroxidase; GTP—guanine triphosphate; HO-1—heme oxygenase-1; IL-6—interleukin-6; iNOS—nitric oxide synthase 2; RAS—rat sarcoma; ROS—reactive oxygen species; SOD—superoxide dismutase; NQO1—NAD(P)H: quinone oxidoreductase 1.

Lee et al. [144] also reported that astaxanthin can inhibit the production of NO, PGE_2_, as well as the expression of pro-inflammatory genes by suppressing the function of NF-κB. Furthermore, astaxanthin also suppressed the activity of the iNOS promoter by inhibiting IKK (IκB kinase) activity. A similar study found that LPS-stimulated mouse neutrophils treated with astaxanthin produce significantly lower levels of the proinflammatory cytokines TNF-α and IL-6. Macedo et al. [145] also report that treatment with 5 mM astaxanthin improved the phagocytic and microbicidal activity of neutrophils. In addition, oxidative damage to lipids and proteins in human neutrophils were significantly lower after astaxanthin treatment. The inhibitory activity of this xanthophyll carotenoid on the secretion of IL-1β, IL-6, and TNF-α has also been observed in U937 (a human lymphoma cell line) cells treated with H_2_O_2_. Cytokine levels in cells pre-incubated with 10 mM astaxanthin before H_2_O_2_ stimulation were significantly lower (less than half) of the levels seen in control cells (cells not pre-treated with astaxanthin). Furthermore, cells pre-incubated with astaxanthin also showed restoration of SHP-1 (a protein tyrosine phosphate) expression levels and reduced levels of NF-*κ*B expression [146]. Bennedsen et al. [147] reported that *Helicobacter pylori*-infected mice fed with astaxanthin extracted from the microalga *H. pluvialis*, showed reduced levels of gastric inflammation. Mice fed with 200 mg/kg of algal extract for 10 days, showed significantly lower levels of inflammation and mucosa-bacterial loads in their stomachs than untreated mice. These results indicate a change in the T-lymphocyte response in mice; the response changes from a predominantly Th1-response to a mixed Th1/Th2-response. This shift was found to occur because of a block in IFN-γ release that boosts IL-4 release in splenocytes in the infected mice pre-treated with astaxanthin. A clinical study to investigate the anti-oxidative and anti-inflammatory effects of astaxanthin was also undertaken on a cohort of healthy young women [148]. In this study, the women who ingested 2 mg of astaxanthin for 8 weeks had lowered blood levels of C-reactive protein, indicating that this compound has anti-inflammatory activity. In addition, the study also found that astaxanthin could reduce ROS production by down-regulating NF-κB and AP-1 transcription factors, as well as inflammatory cytokine production. From these results, it is clear that astaxanthin ingestion can decrease DNA damage, reduce acute phase protein levels, and enhance immune responses in healthy young women [148]. Overall, these studies indicate that astaxanthin inhibits inflammatory processes by blocking the expression of pro-inflammatory genes through suppression of NF-κB activation; a diagrammatic representation of this mechanism is represented in Figure 5.

### 5.6. Anti-Obesity Activity

Obesity is a condition where excessive accumulation and storage of fat in the body occurs, leading to inordinate increases in body weight [149]. Obesity leads to, and exacerbates several conditions, particularly those related to cardiovascular diseases, T2DM, obstructive sleep apnea, certain types of cancer, osteoarthritis, and depression [150]. Since the first half of this century, obesity has been one of the foremost issues of concern regarding public health. In developing countries, increased industrialization has increased the incidence of obesity in teenagers and senior citizens, causing a worrying health trend [151]. Therefore, the search for safe anti-obesity agents is now of great importance.

Wang et al. [152] reported that in obese individuals, there is an excessive accumulation of adipose tissue in organs that have large numbers of fat cells. Obesity is thought to result from adipocyte hypertrophy and the recruitment of new adipocytes from precursor cells. For this reason, the regulation of adipogenesis may be a potential strategy for the treatment of obesity. Okada et al. [153] reported that the chemical structures of carotenoids are important for suppression of adipocyte differentiation; investigations on 13 naturally occurring carotenoids have revealed that molecules with keto or epoxy groups, as well as epoxy-hydroxy carotenoids, hydroxyl-carotenoid, and keto-hydroxy carotenoid have no suppressive effects on adipocyte differentiation. The study found that only fucoxanthin and neoxanthin could significantly suppress adipocyte differentiation, suggesting that the presence of the allenic bond is an important factor for carotenoids to exhibit anti-obesity functions. From these results, it could be hypothesized that carotenoids containing an allenic group and an additional hydroxyl group may be effective in controlling adipocyte differentiation.

**Figure 6:**
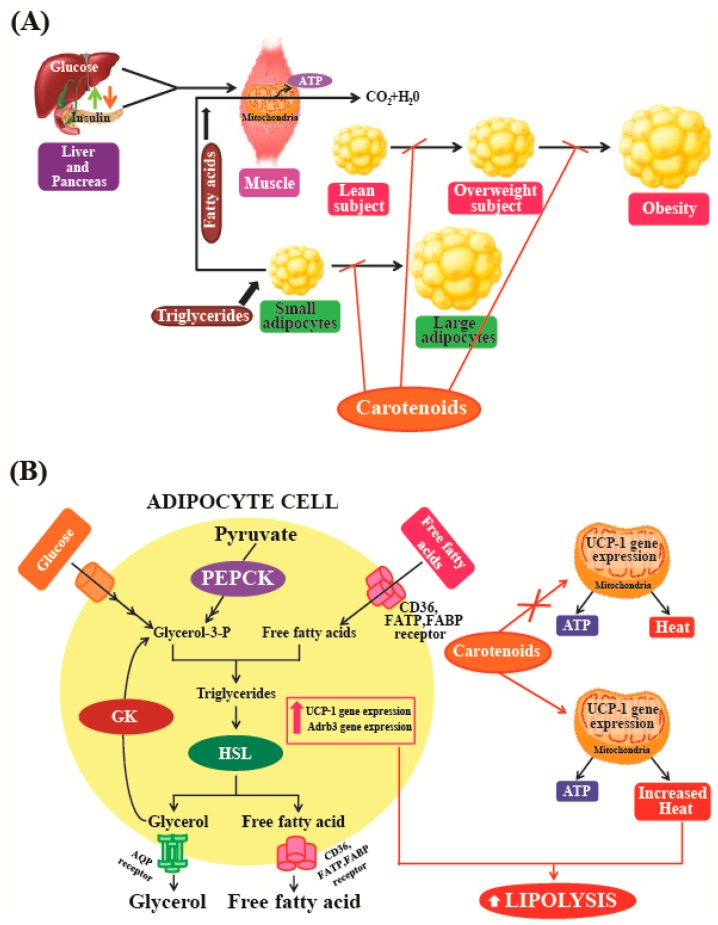
(**A**,**B**) Effects of carotenoid on thermogenesis and lipolysis: the muscle (**A**) and the adipose tissue (**B**). During excess caloric intake in the body, adipocytes take up free fatty acids (FFA), which are stored in the form of triglycerides (TG). For the synthesis of TG other metabolites are required, glycerol-3-phosphate, proceeds from three metabolic sources: (i) glucose; (ii) glycerol derived from lipolysis, which is phosphorylated by glycerol kinase (GK) and (iii) pyruvate, which is converted to glycerol by the activity of phosphoenolpyruvate carboxykinase (PEPCK). During fasting or exercise, TG are hydrolyzed to glycerol and FFA by the hormone-sensitive lipase (HSL) and released into the bloodstream. Several membrane proteins, including fatty acid binding protein (FABP), fatty acid translocase (FAT, CD36) or fatty acid transporter protein (FATP), facilitate the free fatty acid transport across the membrane. At this time, the uncoupling protein 1 (UCP1) and β3-adrenergic receptor (Adrb3) mRNA expression in the abdominal fat tissues and plasma adipokine levels was increased. Carotenoid plays an anti-obesity effect mainly by stimulating uncoupling protein-1 (UCP-1) expression in white adipose tissue (WAT). AQP—aquaporin; CD36—cluster of differentiation 36. ‘X’—indicates that mechanism occur in the absence of carotenoid.

Maeda et al. [154] used a mouse model to show that oral intake of fucoxanthin could significantly decrease the amount of abdominal white adipose tissue (WAT) in obese mice. In addition, the study also found that this treatment had no such effects on normal mice kept on normal diets. This indicates that fucoxanthin specifically suppresses adiposity in obese mice. This study suggests that the anti-obesity effect of fucoxanthin is mediated by alterations in the functioning of lipid-regulating enzymes that could raise plasma adipokine levels and promote higher expression levels of uncoupling protein 1 (UCP1) and β3-adrenergic receptor (Adrb3) in abdominal fat tissues (Figure 6A). UCP1, which is abundant in the inner membrane of the mitochondria, is specifically expressed at high levels in brown adipocytes. UCP1 can dissipate energy by uncoupling the process of oxidative phosphorylation, which then produces heat instead of ATP (Figure 6B). It is well-known that brown adipose tissue (BAT) plays a vital role in the prevention and treatment of obesity [155]. The role of UCP1 in BAT is known to be a significant component of the regulatory system governing whole-body energy expenditure, and the protein is thought to be important in preventing the development of obesity [156]. Increasing UCP1 expression in BAT could be considered as a useful anti-obesity treatment option [157]. However, in humans, most of the body fat is stored in WAT [158]. Furthermore, WAT has now been recognized to function as an endocrine and active secretory organ as its produces biologically active mediators known as adipokines [159]. Fucoxanthin is likely to emerge as an important and attractive anti-obesity agent [94]. However, further studies are needed to clarify the various molecular mechanisms and intracellular signaling pathways that are involved in the anti-obesity activities of fucoxanthin. These studies indicate that natural pigments may play a vital role in the treatment and prevention of obesity, as these molecules may act as regulators of lipid metabolism in fat tissues. Natural pigments obtained from microalgae can be used in functional foods and pharmaceuticals, as these substances can be obtained at relatively low production costs, exhibit low cytotoxicity, and have gained wide acceptance as food supplements. Among the different types of carotenoids, fucoxanthin derived from marine algae may be considered a promising food supplement and weight-loss drug for the prevention and management of obesity.

### 5.7. Anti-Oxidant Activity

ROS and reactive nitrogen species (RNS) are generated during aerobic metabolism processes that occur in the cell; these include processes such as signal transduction, gene expression, and activation of cell signaling cascades [160]. ROS can damage biologically important molecules such as lipids, DNA, and proteins, which can in turn, negatively affect the integrity of cell membrane structures, enzyme functions, and gene expression; ROS are well-known to be involved in the patho-biochemistry of degenerative diseases [161]. The antioxidant defense systems in living organisms are complex networks that are comprised of several enzymatic and non-enzymatic antioxidants [162].

Carotenoids are known to play important roles in scavenging ROS such as singlet molecular oxygen (^1^O_2_) and peroxyl radicals, but there is little information regarding their roles in cellular defenses against RNS. Raposo et al. [13] have reported that the structural features of carotenoids play a significant role in their antioxidant activities. Fucoxanthin extracts from algae show great potential as antioxidants [163]. Fucoxanthin has strong radical-scavenging activity due to the presence of the unusual double allenic bonds at the C-7’ position of its structure, as demonstrated by Sachindra et al. [164]. Miyashita [165] has reported that fucoxanthin can significantly affect human health by altering the gene expression profiles of proteins involved in cell metabolism. Many studies have tested the antioxidant effects of fucoxanthin on different cell lines and animal models [166,167]. Another important carotenoid exhibiting strong antioxidant activity is astaxanthin, which shows higher levels of antioxidant activity than other carotenoids such as β-carotene, zeaxanthin, and canthaxanthin [168]. Rodrigues et al. [169] have reported that astaxanthin acts as a scavenger of various reactive species such as LOO•, HOCl, and ONOO^−^. Several studies have reported that dietary intake of carotenoids can protect humans and animals from oxidative damage to lipophilic parts of cells; this is because carotenoids can limit lipid peroxidation events by scavenging the ROS formed during photo-oxidative processes [13]. To prevent oxidative damage, and disease conditions arising from such damage, a combination of carotenoids possessing different chemical characteristics can be used. Microalgae such as *Spirulina platensis*, *H. pluvialis*, and *Dunaliella salina* might be of great value in the production of various types of such carotenoids (Table 1).

### 5.8. Beauty-Enhancing Effect

Skin has naturally occurring antioxidant agents which can block the effects of ROS and suppress cell disruption and damage [170]. However, when high levels of ROS are produced by ultraviolet (UV)-exposure, these defenses may not provide adequate protection. Apoptosis and necrosis are the two major modes of cell death that occur due to the accumulation of ROS in cells; excessive cell death can lead to wrinkling and dryness of skin. ROS accumulation also plays an important role in photo-aging conditions such as cutaneous inflammation, melanoma, and skin cancer [171]. Natural pigments can be used as therapeutic agents to overcome these problems. As many consumers prefer naturally derived compounds in their cosmetics, there is an increasing global demand for naturally derived carotenoids rather than those synthetized chemically. Due to this demand, the price of natural pigments isolated from algae is roughly double (~700 Euros/kg) of that of synthetic products [172].

Astaxanthin is an excellent antioxidant, exhibiting higher antioxidant activity than vitamins C and E; furthermore, this molecule aids in the preservation of proteins and essential lipids in human lymphocytes as it boosts superoxide dismutase and catalase enzyme activities [12,173]. Tominaga et al. [174] reported that both topical and oral use of astaxanthin can suppress skin hyper-pigmentation, inhibit synthesis of melanin, and improve the condition of all skin layers. Fucoxanthin has been reported to suppress tyrosinase activity in UVB-irradiated guinea pigs, and melanogenesis in UVB-irradiated mice. Studies have also found that oral administration of fucoxanthin decreases the mRNA levels of proteins linked to melanogenesis in skin cells. This indicates that fucoxanthin can negatively regulate melanogenesis factors at the transcriptional level [175]. In addition, fucoxanthin has the ability to counteract oxidative stresses caused by UV radiation, due to which it is currently used in cosmeceuticals [176]. Another important carotenoid exhibiting strong antioxidant activity is β-carotene, which helps in preventing the formation of free radicals that can cause premature aging in skin cells. In the epidermal and dermal layers of skin, the carotenoid lutein has been shown to protect against UV-induced oxidative damage, especially in combination with other antioxidant systems and immunoprotective substances [177].

**Figure 7:**
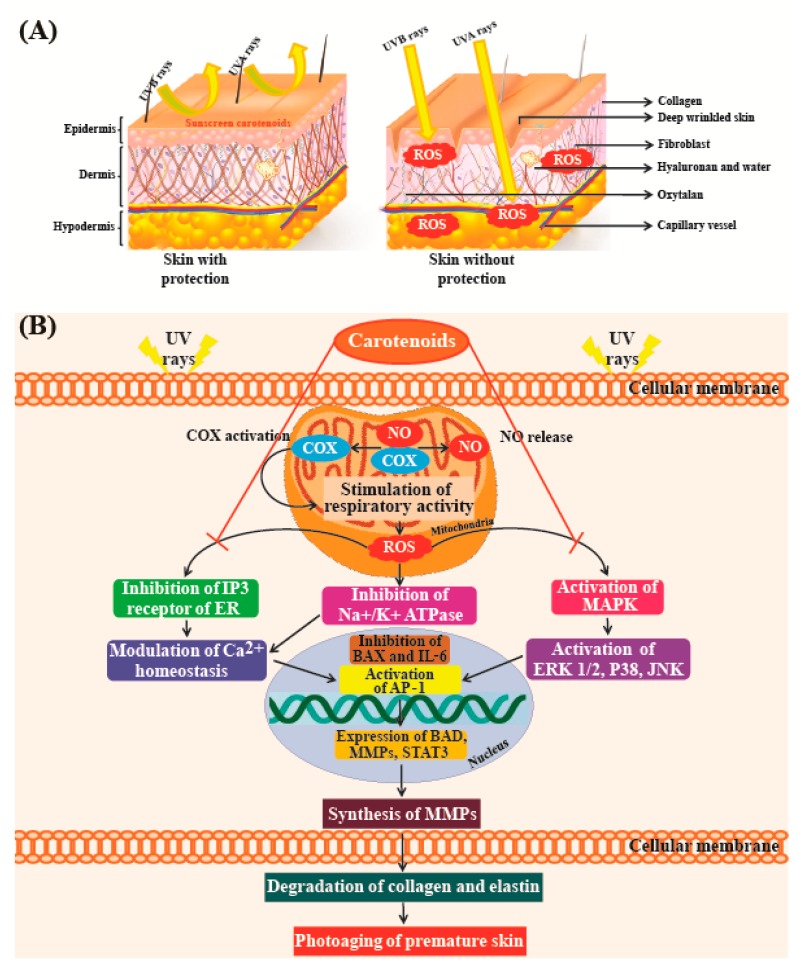
(**A**) Schematic diagram showing penetration of UV rays into the layers of the human skin. UVB rays do not penetrate the skin deeply because they are blocked by the epidermis, whereas UVA penetrate deep into the skin, which leads to damage of elastin and collagen fibers. Carotenoids act as a sunscreen and protect the skin from UV rays; (**B**) Inhibitory effects of UV rays-induced photodamage in premature skins. AP-1—activator protein-1; BAD—BCL2 associated agonist of cell death; BAX—Bcl-2 associated X; COX—cyclooxygenase; ER—endoplasmic reticulum; ERK—extracellular signal-reduced kinase; IL-6—interleukin-6; IP3—inositol 1,4,5-triphosphate; JNK—jun amino-terminal kinase; MAPK—mitogen-activated protein kinase; MMPs—matrix metalloproteinases; NO—nitric acid; ROS—reactive oxygen species; STAT3—signal transducer and activator of transcription 3.

A study conducted by Darvin et al. [178], which compared skin roughness with age in a cohort of women aged 40–50 years, indicated that there was no significant correlation between the two parameters. However, skin roughness was clearly correlated with the concentration of lycopene present in skin. Individuals with higher levels of antioxidants in their skin showed fewer furrows and wrinkles than those with lower levels of antioxidants [178]. Figure 7A shows that UV radiation from the sun is one of the major causes of premature skin aging. Figure 7B shows that UV radiation from sun rays destroys elastin and collagen fibers in the skin [179]. High concentrations of antioxidants such as carotenoids can efficiently neutralize free radicals before they can cause damage. These studies confirm the results of a study conducted by Heinrich et al. [180], which showed that a significant reduction in skin roughness could be achieved with supplements of antioxidant micronutrients such as lycopene.

## 6. Other Health Benefits

### 6.1. Age-Related Macular Degeneration

In older people, age-related macular degeneration (ARMD) is one of the leading causes of visual impairment. In the United States of America, approximately 2.07 million Americans were affected with advanced ARMD in 2010, and this number is expected to increase to 5.44 million in 2050. ARMD is a major cause of irreversible blindness among elderly people (>65 years of age) in western countries, and affects ~20% of the total population [181]. The macula lutea is an oval-shaped pigmented area near the center of the retina, and the area of maximal visual acuity. Recently, the focus of much research has been on investigating the protective effects of carotenoids against ARMD. As carotenoids absorb UV light and other forms of solar radiation that can damage our eyes, these molecules could help in preserving healthy cells in the eyes by reducing oxidative damage and vision loss [181].

An investigation using unilamellar liposomes as a model membrane showed that the filtering effects of lutein and zeaxanthin are higher than those of lycopene and β-carotene. Because of this, lutein and zeaxanthin are thought to be essential pigments in the lens and retina of the eye, and maintaining their levels could be critical in protecting vision in older people [182]. Although the major dietary carotenoids such as α-carotene and lycopene are efficient blue-light filters, these molecules are not found in the macula lutea [183]. The spectral properties of carotenoids, as well as their antioxidant activities can change with the environment. Epidemiological data indicate that macular pigments such as lutein play an important protective role in the eyes [184]. Another study found that the retinas of donors suffering from ARMD had lower levels of lutein and zeaxanthin than those of donors unaffected by ARMD [185]. Furthermore, several reports also indicate that dietary supplementation with lutein alone or lutein together with other nutrients can improve visual function in patients suffering from atrophic ARMD [186]. Stahl and Sies [116], who investigated the combined effects of administering high-doses of β-carotene with vitamin C, vitamin E, and zinc to ARMD patients, found that ARMD progression was slowed and visual acuity improved with this treatment [116].

### 6.2. Neuroprotective Activity

Neuroprotection strategies are defined as the mechanisms and strategies used to protect neuronal cells against injury, apoptosis, dysfunction and/or degeneration in the central nervous system (CNS) by limiting neuronal dysfunction or death after CNS injury [187]. As most synthetic neuroprotective agents have strong side effects, natural bioactive compounds that can act as neuroprotective agents have been under intensive investigation [138]. Many studies have focused on the neuroprotective properties of natural pigments obtained from algae. Okuzumi et al. [188] reported that fucoxanthin can inhibit N-myc expression and cell cycle progression in GOTO cells (a human neuroblastoma cell line). At a concentration of 10 µg/mL, fucoxanthin can significantly inhibit the growth rate of GOTO cells to just 38%, though its exact mode of action remains unclear. Furthermore, the study shows that fucoxanthin can protect cortical neurons from oxidative damage during hypoxia and oxygen reperfusion [189]. Much neuronal damage can occur during re-oxygenation after hypoxia, because re-oxygenation can lead to the generation of significant amounts of ROS. Fucoxanthin exhibits neuroprotective activity mainly because of its ability to scavenge ROS. Based on these reports, carotenoids could be considered as potential neuroprotective agents that can be used to treat or prevent neurodegenerative diseases. So far, most studies on the neuroprotective activities of carotenoids have been carried out in in vitro systems. Therefore, it is vital to conduct in vivo experimental studies to investigate the neuroprotective activities of carotenoids, especially in humans.

### 6.3. Osteo-Protective Activity

Osteoclasts are highly specialized bone cells that break down bone tissues. One of the most recent uses of fucoxanthin has been in the treatment of osteoclast diseases. Das et al. [190] reported that fucoxanthin significantly suppresses the differentiation of RAW264.7 cells. The study found that 2.5 µM of fucoxanthin can activate caspase-3 and induce apoptosis in osteoclast-like cells. These results suggest that fucoxanthin suppresses osteoclastogenesis by inhibiting osteoclast differentiation and by inducing apoptosis in osteoclasts. Furthermore, dietary supplements of fucoxanthin may be useful in preventing bone diseases such as osteoporosis and rheumatoid arthritis, which are known to be related to bone resorption.

### 6.4. Weight Loss

Fucoxanthin is a well-known weight-loss agent that encourages the ‘burning of fats’ by enhancing thermogenin expression. As fucoxanthin has numerous health benefits, and the molecule is found in high concentrations in microalgae, industrial production of fucoxanthin from microalgae is on the rise [40]. In 2009, a double-blind, randomized, and placebo-controlled study on human volunteers showed that ingesting 2.4 mg of fucoxanthin can lead to significant weight loss. Women ingesting fucoxanthin also exhibited higher levels of resting energy expenditure, which helps in reducing fat and body weight. Furthermore, fucoxanthin consumption also led to significant reductions in blood pressure, and levels of liver fat, triglycerides, and C-reactive protein [191].

## 7. Conclusions and Future Direction of Research

Microalgae are rich sources of carotenoids, with great industrial potential and accessibility, and thus are likely to have a wide range of applications in the healthcare and cosmetic industries. Most studies on carotenoids have been focused mainly on the preventive and protective effects of these molecules in various chronic diseases such as diabetes mellitus, metabolic syndrome, cancer, and cardiovascular diseases. Recent studies, however, report that carotenoids might also play a significant role in the treatment of various other diseases. Although the mechanisms of antioxidant activity for some carotenoids have been well studied, most of the other effects of carotenoids, such as their pro-vitamin A activity, metabolic activity, effects on the immune and endocrine systems, as well as their effects on cell cycle regulation, apoptosis, and cell differentiation have not yet been studied in detail. Although there are a number of ongoing studies investigating the use of carotenoids to enhance healthcare and beauty, most of these studies have been carried out in animal models, with very few human clinical trials. Future areas of research will need to focus on human clinical trials. In addition, these studies must collect detailed data on subject selection, end point measurements and levels of carotenoids being tested. It is hoped that such studies, will help researchers understand the roles and potential uses of carotenoids in developing new strategies for the prevention, treatment, and management of diseases.

## Figures and Tables

**Table 1:** Carotenoids produced by microalgae.

Main Carotenoid	Microalgae	Other Carotenoids	Concentration	General Uses	References
Astaxanthin	*Haematococcus pluvialis*	β-carotene, Lutein, Canthaxanthin, Neoxanthin, Violaxanthin, Zeaxanthin, Echinenone	Up to 7% DW; 75% TC	In benign prostatic hyperplasia and prostate and liver tumors Anti–inflammatory properties Active against liver neoplasms Strong anti-oxidant property Cardiovascular health	[13,16,17,18,19,20]
	*Chlorella zofingiensis*	-	3.7% DW		[21]
β-carotene	*Chlorella zofingiensis*	Canthaxanthin (97% DW), Astaxanthin (0.7% DW)	0.9% DW	Provitamin A function In colorectal cancer In the prevention of acute and chronic coronary syndromes Photoprotection of skin against UV light Prevent night blindness Anti-oxidant property Prevents liver fibrosis	[3,4,13,22,23,24,25,26,27,28,29,30,31,32]
	*Dunaliella salina*	Zeaxanthin, Lutein, α-carotene	10–13% DW		
	*Spirulina maxima*	Astaxanthin, Lutein, β-cryptoxanthin, Zeaxanthin, Echinenone, Oscillaxanthin, Myxoxanthophyll	80% TC		
Canthaxanthin	*Coelastrella striolata* var. *multistriata*	Astaxanthin (0.15% DW), β-carotene (0.7% DW)	4.75% DW	Create a tan color Anti-oxidant property	[33]
Canthaxanthin and Lutein	*Chlorella vulgaris*	Astaxanthin 12.5% TC Violaxanthin	45% TC	Create a tan color Anti-oxidant property In the prevention of acute and chronic coronary syndromes and stroke In the prevention of cataracts To prevent macular degeneration associated with age In the prevention of retinitis To avoid gastric infection by *H. pylori*	[34,35]
Echinenone	*Botryococcus braunii*	Botryoxanthins A and B—0.03% DW Braunixanthins 1 and 2—0.06% DW	0.17% DW	-	[36]
Fucoxanthin	*Cyclotella* cf. *cryptica*	-	0.7 mg g^−1^	Anti-obesity Anti-oxidant property	[37]
	*Cyclotella meneghiniana*	-	2.3 mg g^−1^		[37]
	*Cylindrotheca closterium*	-	0.52% DW		[38]
	*Isochrysis* aff. *galbana*	-	1.8% DW		[39]
	*Mallomonas* sp. SBV13	-	26.6 mg g^−1^		[37]
	*Nitzschia* cf. *carinospeciosa*	-	5.5 mg g^−1^		[37]
	*Odontella aurita*	Diadinoxanthin, β-carotene	up to 2.2% DW		[40]
	*Paralia longispina*	-	1.4 mg g^−1^		[37]
	*Phaeodactylum tricornutum*	Diadinoxanthin, Zeaxanthin, Neoxanthin, Violaxanthin, β-carotene	1.65% DW		[39,41,42]
		-	10.2 mg g^−1^		[37]
Lutein	*Auxenochlorella protothecoides*	Astaxanthin	0.76 mg g^−1^	In the prevention of acute and chronic coronary syndromes and stroke Helps to maintain a normal visual function In the prevention of cataracts To prevent macular degeneration associated with age In the prevention of retinitis To avoid gastric infection by *H. Pylori* Anti-oxidant property Anti-cancer activity	[43]
	*Chlorella protothecoides*	-	5.4 mg g^−1^		[44]
	*Chlorella pyrenoidosa*	Violaxanthin, Loroxanthin, α- and β-carotene	0.2–0.4% DW		[13,18,25,45,46,47]
	*Chlorella sorokiniana*	Astaxanthin	5.90 mg g^−1^		[43]
	*Chlorella* sp.	Astaxanthin	2.26 mg g^−1^		[43]
	*Coelastrella* sp.	Astaxanthin	6.49 mg g^−1^		[43]
	*Galdieria sulphuraria*	-	0.4 mg g^−1^		[48]
	*Parachlorella kessleri*	Astaxanthin	0.28 mg g^−1^		[43]
	*Scenedesmus almeriensis*	-	0.54% DW		[49]
	*Scenedesmus bijugus*	Astaxanthin	2.9 mg g^−1^		[43]
	*Scenedesmus* sp.	Astaxanthin	1.8 mg g^−1^		[43]
	*Vischeria stellata*	Astaxanthin	1.50 mg g^−1^		[43]
Violaxanthin	*Chlorella ellipsodea*	Antheraxanthin, Zeaxanthin	-	Anti-inflammatory activity	[35,50]
Zeaxanthin	*Porphyridium cruentum*	β-carotene	97.4% TC	In the prevention of acute and chronic coronary syndromes Helps to maintain a normal visual function In the prevention of cataracts To prevent macular degeneration associated with age	[13,19,25,46,47,51]

DW—Dry weight; TC—Total carotenoids; ‘-’—No data available.
